# Culturable halophilic archaea at the initial and crystallization stages of salt production in a natural solar saltern of Goa, India

**DOI:** 10.1186/2046-9063-8-15

**Published:** 2012-06-29

**Authors:** Kabilan Mani, Bhakti B Salgaonkar, Judith M Braganca

**Affiliations:** 1 Department of Biological Sciences, BITS PILANI, K K Birla Goa Campus, NH 17 B, Zuarinagar, Sancoale, Goa 403 726, India

**Keywords:** Archaea, Haloarchaea, Hypersaline, Solar saltern

## Abstract

**Background:**

Goa is a coastal state in India and salt making is being practiced for many years. This investigation aimed in determining the culturable haloarchaeal diversity during two different phases of salt production in a natural solar saltern of Ribandar, Goa. Water and sediment samples were collected from the saltern during pre-salt harvesting phase and salt harvesting phase. Salinity and pH of the sampling site was determined. Isolates were obtained by plating of the samples on complex and synthetic haloarchaeal media. Morphology of the isolates was determined using Gram staining and electron microscopy. Response of cells to distilled water was studied spectrophotometrically at 600nm. Molecular identification of the isolates was performed by sequencing the 16S rRNA.

**Results:**

Salinity of salt pans varied from 3-4% (non-salt production phase) to 30% (salt production phase) and pH varied from 7.0-8.0. Seven haloarchaeal strains were isolated from water and sediment samples during non-salt production phase and seventeen haloarchaeal strains were isolated during the salt production phase. All the strains stained uniformly Gram negative. The orange-red acetone extract of the pigments showed similar spectrophotometric profile with absorption maxima at 393, 474, 501 and 535 nm. All isolates obtained from the salt dilute phase were grouped within the genus *Halococcus*. This was validated using both total lipid profiling and 16S rRNA data sequencing. The isolates obtained from pre-salt harvesting phase were resistant to lysis. 16S rRNA data showed that organisms belonging to *Halorubrum*, *Haloarcula*, *Haloferax* and *Halococcus* genera were obtained during the salt concentrated phase. The isolates obtained from salt harvesting phase showed varied lysis on suspension in distilled water and /or 3.5% NaCl.

**Conclusion:**

Salterns in Goa are transiently operated during post monsoon season from January to May. During the pre-salt harvesting phase, all the isolates obtained belonged to *Halococcus sp*. During the salt harvesting phase, isolates belonging to *Halorubrum*, *Haloarcula*, *Haloferax* and *Halococcus* genera were obtained. This study clearly indicates that *Halococcus sp.* dominates during the low salinity conditions.

## Findings

Marine solar salterns are thalassohaline hypersaline environments located in tropical and subtropical areas worldwide, consisting of shallow ponds for the production of common salt from seawater during summer. The method of making salt through natural evaporation dates back to pre-historic times. This traditional approach of salt production involves construction of series of rectangular ponds, each connected to the other through a common opening [1-4].

Goa (15°34′60N, 74°0′0E) is a coastal state in India and salt making is being practiced for many years. Saltpans are found in Pernem, Bardez, Tiswadi and Salcete talukas of Goa. Saltpans are inundated by sea water from estuaries during high tides. Sea water is retained in every pond for certain time to facilitate evaporation. As concentration of NaCl gradually increases, first component to precipitate is calcium ion (Ca^2+^) in the form of gypsum. Then the concentrated sea water is allowed into final crystallizer pond, where NaCl crystals precipitate out [1]. The whole process of concentrating sea water begins usually in January - February and salt crystals are harvested during March - May. During the remaining months of June - December, ponds are inundated by sea/rain water. The crude salt produced from these saltpans is being used domestically for cooking, ice plants, as fertilizers, as termite repellent and for curing dry fish.

Microbial life is found in various extreme environments and salt pans are no exception. Depending on salt concentration, salt pans are inhabited by different groups of microbes thriving symbiotically [5]. Various microbes that inhabit the salt pans range from prokaryotes like Bacteria (*Salinibacter* spp*.*) and Archaea (*Halobacterium* spp*.*) to eukaryotes like Fungi (*Hortaea* spp*.*) and Algae (*Dunaliella* spp*.*) [6,7].

Haloarchaea are a group of extreme halophiles which require at least 2.5 M NaCl for their growth and are placed in the order *Halobacteriales* under family *Halobacteriaceae*[8]. At the time of writing, family *Halobacteriaceae* accommodated 36 recognized genera, members of which inhabiting both thalassohaline and athalassohaline environments [9,10]. Many studies have shown that there is a great variation in the diversity and dominance of haloarchaeal genera within various geographical locations [11-16]. Novel haloarchaeal microorganisms have been isolated from various econiches such as acidic and alkaline regions, animal hides, salted fishes and also from commercial salt [17-23].

The aim of the present investigation was to evaluate the diversity of culturable haloarchaeal members in saltpans of Goa during two stages of salt production. For this investigation, the diversity of haloarchaea found within a single solar saltern at Ribandar (15°30′N, 73°51′E), Goa was examined.

**Figure 1:**
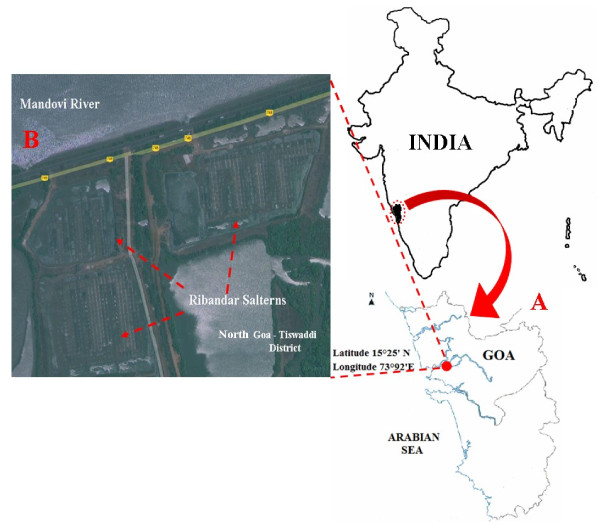
Solar Salterns of Ribandar, Goa. A) Location of Ribandar Salterns in Goa. B) Aerial image of the Ribandar Salterns. [source: Google Earth, 2010].

Ribandar salterns are located on the banks of the river Mandovi, surrounded by mangrove vegetation in the adjoining marshy area. These salt pans cover an area about 12 dm^2^ and lie between the cities of Panaji and Old Goa (Figure 1). Ribandar experiences tropical monsoon climate with maximum temperature around 30-36°C in summer and minimum around 20-28°C in winter. This region receives a heavy monsoon rainfall averaging around 300 cm. The salt pans are located at 9 ft above sea level and experience strong coastal winds during summer facilitating the evaporation of water in salterns. The salt pans are surrounded by raised mud borders called as *bunds* (dykes). These *bunds* help in containing the sea water within the pan. The sluice gate at the inlet regulates the inflow of water from the Mandovi estuary ( Additional file 1: Figure S1, supplementary data). The salterns are seeded with crude salt to speed up the crystallization process. During April and May the salt is harvested daily from these salterns.

Water samples and sediment samples were collected from 0-10 cm distance from the surface Sampling was carried out twice during the two phases of salt production. First sampling was carried out in February 2010, when the saltpans are full of sea water. The second sampling was carried out in April 2010, when salt harvesting is at its peak. Water and sediment samples were collected by scooping from the surface as well as at a depth of 10 cm. Salinity and pH of sampling sites were measured using conductivity meter (EQUIP-TRONICS MODEL EQ-682) and pH meter (EQUIP-TRONICS MODEL EQ-632). Conductivity was correlated to salinity using the equation given by Williams, 1986 [24].

(1) $$\text{S}=0.4665\times^{1.0878}\left({\text{r}}^{2}=0.98799\right)\;$$

The samples were collected in sterile tubes, sterile 1 L bottles and stored at 4°C and processed within 48 h.

Direct plating and enrichment techniques were employed for isolation of haloarchaeal organisms from water and sediment samples. Two media were used in the study, NaCl Tryptone Yeast Extract (NTYE) medium and NaCl Tri-Na-citrate (NT) medium [25-27], both containing 25% NaCl. The main difference with both the media is the presence of trisodium citrate in NT medium, which can support the growth of fastidious organisms as compared to NTYE medium. In direct plating method, one hundred μl of water sample or a loopful of sediment sample was directly spread plated on media. In the enrichment technique method, one ml of water sample or one gram of sediment sample was aseptically transferred to 50ml media and incubated at 37°C for up to 5 days. Then ten μl aliquots were plated on media. Plates were incubated at room temperature (30°C) for 30 to 45 days until red-orange pigmented colonies appeared. Colonies were selected based on their morphology and/or pigmentation and purified through repeated sub-culturing.

Morphology of the isolates was determined using Gram staining and electron microscopy. Cell suspensions were prepared on glass slides in a drop of 15% (w/v) NaCl solution and air-dried. The cells were desalted with 2% acetic acid followed by Gram staining and observed using phase-contrast microscope (Olympus BX41). For electron microscopy, the cell pellets were dispensed in NaCl Synthetic Media (NSM) to an absorbance of 0.8 at 600nm [28]. One hundred μl of suspension was mounted onto circular glass cover slips, fixed with 2.0% glutaraldehyde fixative (prepared in NSM) at room temperature (30°C) overnight. The coverslips were then exposed, to a series of increasing gradient of acetone-water, corresponding to 30%, 50%, 70%, 90% for 10 min respectively and finally in 100% acetone, for 30 min, air dried and then viewed under scanning electron microscope (JEOL-5800 LV SEM).

Pigments were extracted by sonicating the cells for 30 min at a pulse rate of 0.5 s in acetone or chloroform: methanol (2:1). Identification of pigment was done through spectrophotometric scans in both UV and visible range (190-800nm). Total polar lipids were analysed as described by Litchfield et al in 2000 and Oren et al in 1996 [29,30].

Response of cells to distilled water was studied spectrophotometrically at 600nm (Shimadzu, Japan). Cell viability assay was performed by resuspending the cells in distilled water, 3.5% NaCl and 30% NaCl and then incubating them for time periods of 24 h to 10 days respectively [31]. The cell suspensions were then plated and observed for viable colony formation.

Molecular characterization of the isolates was performed by extracting genomic DNA using phenol-chloroform method and amplifying gene for 16S rRNA with primers A109 (F) AC(G/T)GCTCAGTAACACGT and 1510(R) GGTTACCTTGTTACGACTT [32]. Each PCR reaction contained 2 U Taq Polymerase, 10X Taq buffer, 2 mM MgCl_2,_ 10 mM of dNTPs (Sigma), 10 μM of each primer (IDT technologies) and 1 μl of template DNA. Final reaction was made up to 50 μl with ultra-pure distilled water. The amplification was performed under the following conditions: Initial denaturation for 5 min at 94°C, denaturation for 30 s at 94°C, annealing for 40 s at 53.5°C, elongation for 60 s at 68°C (35 cycles) and final elongation at 68°C for 5 min. Amplified products were purified and then sequenced using an automated DNA sequencer (Applied Biosystems). The sequencing results of the amplified 16S rRNA fragments were subjected to BLAST analysis. Multiple sequence alignment was done out using MUSCLE and phylogenetic tree was constructed with MEGA 5.0 by neighbor-joining method with bootstrap analysis using 1000 replicates [33,34].

Specific conductance (conductivity) is a measure of the electrical current of a solution. The greater the salinity, greater is the conductivity. Salinity of salt pans varied from 3-4% (non-salt production phase) to 30% (salt production phase) and pH varied from 7.0-8.0. To determine the nature of elements contributing to the salinity, total chemical analysis of the brine water revealed that Na^+^ and Cl^-^ are the dominant ions. This indicates the thalassohaline nature of the brine. The main cations were Na^+^ (747 g/l), Ca^2+^ (121 g/l), K^+^ (80 g/l) and Mg^2+^ (171 g/l) and the main anion was Cl^-^ (611 g/l).

Plating of sediments and/or water samples on NTYE agar plates, resulted in 30 cream/yellow/white and 14 orange/red colored colonies during the salt dilute phase and about 60-920 cream/yellow/white and 70 -110 orange/red colonies during salt harvesting phase, on incubation of the plates for 20-30 days at room temperature (30°C). On NT medium during pre-salt harvesting phase matt growth of cream/yellow/white and pale pink colonies were obtained. During salt harvesting phase about 20 cream colonies appeared within 24 h of incubation. On further incubation of NT media plates for 8-20 days, light orange, orange red to brick red pigmented colonies appeared. Some of the orange-red colonies growing on NTYE and NT plates were accompanied by white/cream colonies, which persisted even after several subcultures. However these white/cream colonies were eliminated when streaked on NTYE and NT plates containing 50 μg/ml of ampicillin, indicating their bacterial origin ( Additional file 1: Figure S2, supplementary data).

Since carotenoid or bacterioruberin pigment is one of the characteristic features of haloarchaea, orange-red colored colonies were selected on basis of their difference in colony morphology and pigmentation.

During the pre-salt harvesting phase, orange-red colonies appeared after 20 -30 days of incubation. These colonies appeared as a uniform pure culture in all the water/sediment samples plated. However based on visual differentiation seven different types of orange-red isolates were picked from NTYE medium and were designated as BK3, BK6, BK7, BK11, BK18, BK19 and BK20. The isolates were maintained on NTYE agar slopes.

During the salt harvesting phase, fifteen visually different pigmented isolates were obtained on NT agar medium and were designated as BS1, BS2, BS3, BS4, BS5, BS6, BS7, BS8, BS11, BS13, BS15, BS16, BS17, BS19 and BS20. Two strains BBK1and BBK2, orange/red in color, were isolated from NTYE agar medium (Table 1).

**Table 1:** Halophilic archaeal isolates obtained from Ribandar solar salterns of Goa, India

**Saltern phase and econiche**	**Isolates**	**Pigmentation**	**Gram character and morphology**	**Lysis in**		**Identification**	**Accession No.**
				**Distilled water**	**3.5% salt solution**		
**Pre salt harvesting phase/Initial Stage/Salt Dilute Stage**							
Water samples	BK3	Bright Orange-red	Gram negative cocci	-	-	*Halococcus salifodinae*	HQ455793
	BK6	Bright Orange-red	Gram negative cocci	-	-	*Halococcus salifodinae*	AB588757
	BK7	Bright Orange-red	Gram negative cocci	-	-	*Halococcus salifodinae*	HQ455794
	BK11	Bright Orange-red	Gram negative cocci	-	-	*Halococcus salifodinae*	HQ455795
Sediment samples	BK18	Orange	Gram negative cocci	-	-	*Halococcus salifodinae*	HQ455796
	BK19	Bright Orange-red	Gram negative cocci	-	-	*Halococcus salifodinae*	AB588758
	BK20	Light Orange	Gram negative cocci	-	-	Not sequenced	Not sequenced
**Salt harvesting phase/Crystallization Stage**							
Brine samples	BBK1	Orange	Gram negative cocci	-	-	*Halococcus salifodinae*	AB588755
	BBK2	Orange	Gram negative cocci	+	+/-	*Haloferax volcanii*	AB588756
	BS1	Bright red	Gram negative cocci	+	+/-	*Haloarcula argentinensis*	HQ455797
	BS2	Bright red	Gram negative cocci	+	+/-	*Haloarcula japonica*	HQ455798
	BS3	Bright red	Gram negative cocci	+	+/-	*Haloarcula sp.*	HQ455799
	BS5	Bright red	Gram negative pleomorphic	+	+/-	*Haloarcula argentinensis*	AB588759
	BS6	Bright red	Gram negative short rods	+	+/-	*Haloarcula hispanica*	HQ455801
	BS7	Bright red	Gram negative pleomorphic	+	+/-	*Haloarcula japonica*	HQ455802
Sediment samples	BS17	Bright red	Gram negative cocci	+	+/-	*Halorubrum sp.*	ND
	BS16	Light Orange	Gram negative cocci	+	+/-	*Haloferax alexandrinus*	HQ455803

“-” No Lysis; “+” Lysis; “ND” Not Deposited.

**Figure 2:**
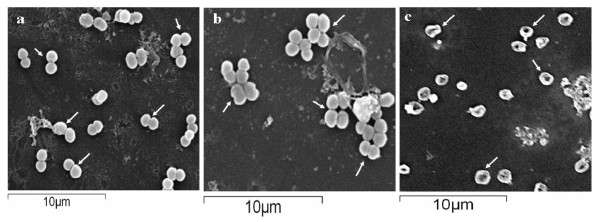
**Scanning electron micrograph of Haloarchaeal isolates a) BK3, b) BK19 and c) BBK2 grown in NTYE liquid medium.** (*Bar*, 10μm).

All the strains stained uniformly Gram negative. All of the BK and BBK series cultures appeared as cocci either as singles, pairs, chains or groups. Cultures in BS series (BS1, BS2, BS3, BS5, BS6, BS7 BS13, BS15 and BS20) appeared as tiny cocci whereas cultures BS4, BS11, BS17 and BS19 appeared as short rods. The coccoid morphology of the cultures was further confirmed by scanning electron microscopy. Most of the coccoid isolates appeared as single cells or diplococci with exception of BK19 which exhibited classical *Sarcina* like packets. The isolates BBK2 and BS16 exhibited unique morphology which appeared as flattened involuted discs (Figure 2).

The orange-red acetone or chloroform: methanol extract of the pigment showed similar spectrophotometric profile with absorption maxima at 393, 474, 501 and 535 nm. These peaks correspond to bacterioruberin pigments which are typical pigments of haloarchaea [35,36].

**Figure 3:**
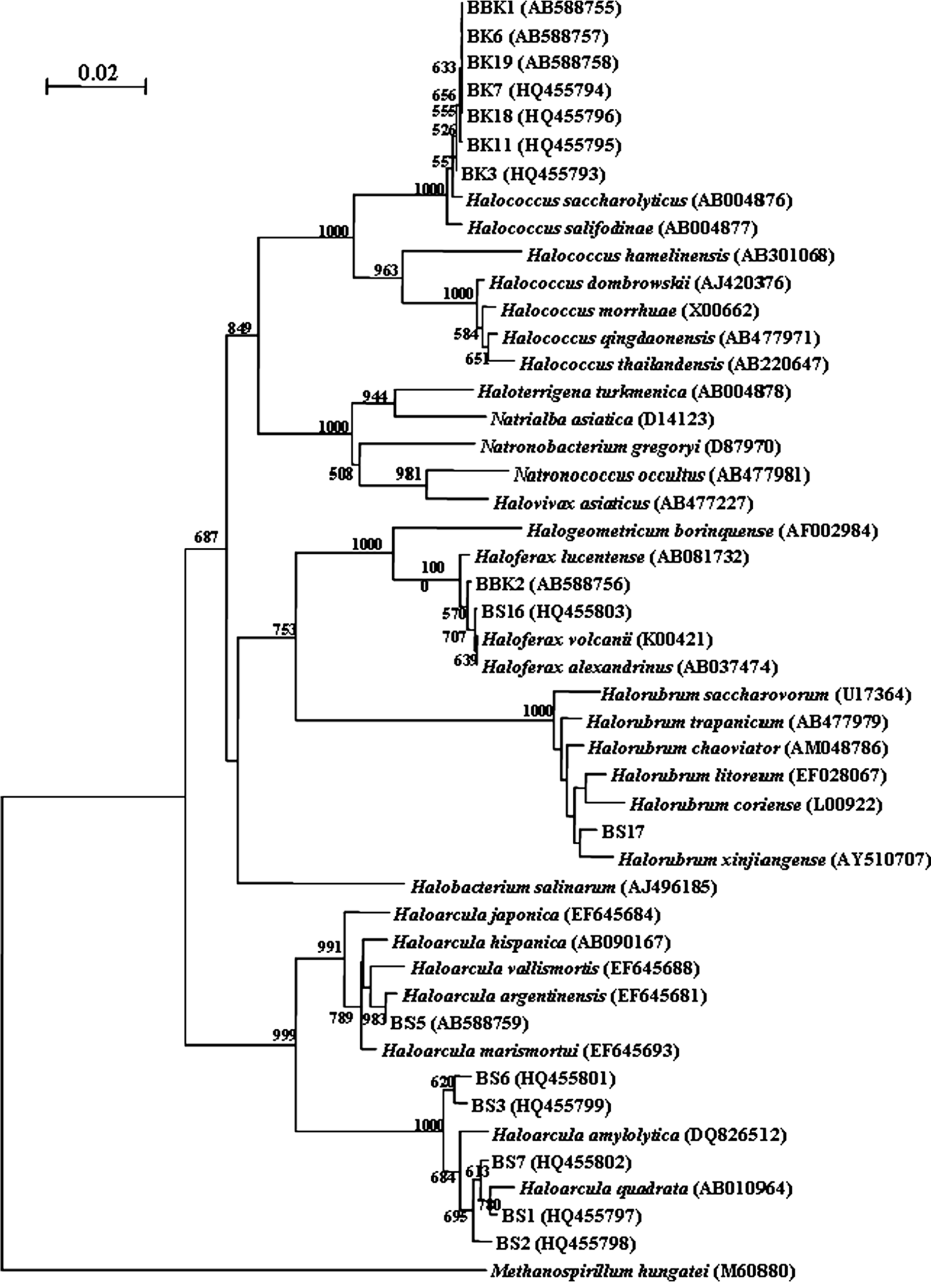
**Phylogenetic tree showing the positions of isolated haloarchaeal strains constructed with MEGA 5.0 by using NJ method.***Methanospirillum hungatei* was used as an outgroup.

The BLAST analysis of the 16S rRNA gene fragments of the six isolates BK3, BK6, BK7, BK11, BK18 and BK19 obtained from the pre-salt harvesting phase showed 98-99% similarity to Halococcus salifodinae and Halococcus saccharolyticus (Figure 3). Among isolates from salt the harvesting phase, only BBK1 (AB588755) was very close to *Halococcus sp.* with 98-99% similarity whereas cultures BS1, BS2, BS3, BS5, BS6 and BS7 were assigned to the genera *Haloarcula* with similarity of 98-99%. BBK2 and BS16 showed highest similarity to *Haloferax alexandrinus of* about 97-98% and the 16S rRNA sequence of BS17 and BS19 was closely related to the genera *Halorubrum* with 98% similarity.

As noted earlier, orange – red colonies from pre-salt harvesting phase were almost uniform and appeared to be in pure form. All these isolates showed 99% similarity to the genera *Halococcus*. It was of interest to see if there were any differences in these strains. Hence the total polar lipids were studied. The lipid profile obtained showed 2 spots of phosphotidylglycerol (PG), methyl ester of phosphatidyl glycerophosphate (PGP-Me) corresponding to R_f_ values 0.828 and 0.517 respectively which are the signature polar lipids of haloarchaea. The glycolipid, sulfated diglycosyl diether lipids (S-DGD) having an R_f_ of 0.368 was seen in all the strains of BK and BBK series which is typical of genus *Halococcus* and *Haloferax*[37]. No differentiation at the strain level could be done based on the lipid profile. Most likely it was the same strain that had been isolated multiple times, due to its abundance in the environment.

 The isolates obtained from pre-salt harvesting phase (BK series) were resistant to lysis in distilled water and 3.5% NaCl. However isolates obtained from salt harvesting phase showed varied lysis on suspension in distilled water with the exception of BBK1 which was resistant to lysis. The isolate BBK2 lysed immediately, where as BS4 and BS5 were observed to have delayed lysis. Viability assay was performed for BK6 obtained during pre-salt harvesting phase and BBK2 obtained during salt harvesting phase. On suspension of cells in distilled water, 3.5% NaCl and 30% NaCl, followed by plating revealed that isolate BK6 was viable even after 10 days on suspension in distilled water. Cells of BK6 also retained their coccoid shape, when observed microscopically. The isolate BBK2 survived for up to 24 h in 3.5% NaCl, but lysed immediately in distilled water showing no growth on plating.

Interestingly the isolates of the BK series failed to lyse in acetone for extraction of pigment even on sonication but lysed on suspension in Chloroform : methanol (2:1 v/v).

It is interesting to see that Halococci were recovered almost as a pure culture in culturable form during the salt dilute phase (whereas other culturable genera could be recovered during the salt concentrated phase). Recent studies have shown that haloarchaea are being isolated from less hypersaline environments. Salt-marsh sediments, sulfur-rich spring and deteriorated ancient wall paintings has been investigated and found to have haloarchaeal members, predominantly belonging to the genera *HalococcusHaloferax* and *Halogeometricum*[38-41]. A study by Fukushima et al. (2007), showed that the cells of *Halococcus* survived in sea water (salinity of which is 3.5%) upto 9 days without losing its cell rigidity. It is also possible that haloarchaea are trapped in the salt crystals and get deposited in the sediments. The salinity of sediments obtained at about 10 cm was 6-10%. Therefore it is quite possible that even though the haloarchaeal members cannot flourish they can still retain their viability. Another possibility is presence of clay in these salterns. These clay particles have micropores on which the salt fluid gets filled along with the haloarchaeal members. These micropores could serve as a salt rich environment for the survival of haloarchaea [42-44]. Diversity studies of hypersaline areas around the world have indicated that *Halorubrum* and *Haloarcula* are the dominant culturable members of haloarchaea [45-50]. This investigation revealed that haloarchaeal members belonging to the genus *Halococcus,* thrive in less saline environments and are the dominant culturable haloarchaea during the pre-salt harvesting phase or the salt dilute phase.

The salt pan under study is transient and operated only during the post monsoon season of January to May. During monsoons, the saltpans are inundated with sea water as well as rain water. Most of haloarchaeal strains are known to lyse in distilled water as they require at least 10% NaCl to maintain the integrity of their outer membrane. It is interesting to note that Halococcal microorganisms were most abundant, during the pre-salt harvesting phase as they are resistant to lysis in lowering salinities than their counterparts. However, as salinity gradually increases, other members of haloarchaeal community start to colonize the saltpans. Of the thirty six defined genera in the family *Halobacteriaceae*, four different genera namely, *Halococcus*, *Haloferax*, *Haloarcula* and *Halorubrum* were found to be represented in this study. This investigation provides valuable information about the change in culturable haloarchaeal diversity under variable salt conditions.

Nucleotide sequence data can be accessed from DDBJ and NCBI database under the accession numbers HQ455793 - HQ455803 and AB588755 - AB588759.

## Competing interests

The authors declare that they have no competing interests.

## Authors’ contributions

JB conceived the idea and designed the study. KM, BBS and JB conducted the field operations. KM carried out the laboratory work of physico-chemical analysis of the samples, molecular characterisation of the isolates and drafted the manuscript. BBS isolated the haloarchaeal strains and performed pigment characterisation and cell viability assays. KM and BBS carried out the SEM studies. JB edited and revised the manuscript. All authors have read and approved the final manuscript.

## Supplementary Material

Additional file 1 **Figure S1.** Solar Salt production at the Ribandar salterns a) bed preparative stage showing series of rectangular beds (January) b) Sluice gate / inlet point for entry of saline water c-d) rectangular beds inundated with saline water (February - March) e) tool used for extracting salt f) crude salt heaped up at the corners of the *bandhs* g) collection of brine sample h) crude salt collected and piled up on the sides i) collection of sediment sample. Figure S2. Media plates (NTYE and NT) showing diversity of halophilic microorganisms obtained during initial stage and crystallization stages of salt production. Interested bright orange culture obtained on media containing ampicillin. Click here for file

## References

[B1] Javor BJ Industrial microbiology of solar salt production J Ind Microbiol Biotechnol 2002 28 42 47 11938470 10.1038/sj/jim/7000173

[B2] Hoffman BA Dawes CJ Vegetational and Abiotic Analysis of the Salterns of Mangals and Salt Marshes of the West Coast of Florida J Coastal Res 1997 13 147 154

[B3] Rodrigues CM Bio A Amat F Vieira F Artisanal salt production in Aveiro/Portugal - an ecofriendly process Saline Syst 2011 7 3 10.1186/1746-1448-7-3 22053788 PMC3225316

[B4] Rocha RM Costa DFS Lucena-Filho MA Bezerra RM Medeiros DHM Azevedo-Silva AM Araújo CN Lauro Xavier-Filho L Brazilian solar saltworks - ancient uses and future possibilities Aqua Biosyst 2012 8 8 10.1186/2046-9063-8-8 PMC3349464 22490329

[B5] Bardavid RE Khristo P Oren A Interrelationships between Dunaliella and halophilic prokaryotes in saltern crystallizer ponds Extremophiles 2008 12 5 14 10.1007/s00792-006-0053-y 17186316

[B6] Oren A The ecology of the extremely halophilic archaea FEMS Microbiol Rev 1994 13 415 439 10.1111/j.1574-6976.1994.tb00060.x 19709178

[B7] Oren A Microbial life at high salt concentrations: phylogenetic and metabolic diversity Saline Syst 2008 4 2 10.1186/1746-1448-4-2 PMC2329653 18412960

[B8] Grant WD Kamekura M McGenity TJ Ventosa A Boone DR, Castenholz RW, Garrity GM Order I.Halobacteriales Grant and Larsen 1989b, 495VP Bergey´s manual of systematic bacteriology 2001 2 Springer, New York 294 301

[B9] Makhdoumi-Kakhki A Amoozegar MA Bagheri M Ramezani M Ventosa A Haloarchaeobius iranensis gen. nov., sp. nov., an extremely halophilic archaeon isolated from the saline lake Aran-Bidgol, Iran Int J Syst Evol Microbiol 2011 62 1021 1026 21685256 10.1099/ijs.0.033167-0

[B10] Oren A Taxonomy of the family Halobacteriaceae: a paradigm for changing concepts in prokaryote systematics Int J Syst Evol Microbiol 2012 62 263 271 10.1099/ijs.0.038653-0 22155757

[B11] Ahmad N Sharma S Khan FG Kumar R Johri S Abdin MZ Qazi GN Phylogenetic analyses of Archaeal Ribosomal DNA sequences from salt pan sediment of Mumbai, India Curr Microbiol 2008 57 145 152 10.1007/s00284-008-9167-z 18543030

[B12] Manikandan M Kannan V Pasic L Diversity of microorganisms in solar salterns of Tamil Nadu, India World J Microbiol Biotechnol 2009 25 1001 1017

[B13] Munson MA Nedwell DB Embley TM Phylogenetic diversity of Archaea in sediment samples from a coastal salt marsh Appl Environ Microbiol 1997 63 4729 4733 9406392 10.1128/aem.63.12.4729-4733.1997 PMC168796

[B14] Ochsenreiter T Pfeiffer F Schleper C Diversity of Archaea in hypersaline environments characterized by molecular-phylogenetic and cultivation studies Extremophiles 2002 6 267 274 10.1007/s00792-001-0253-4 12215811

[B15] Oh D Porter K Russ B Burns D Dyall-Smith M Diversity of Haloquadratum and other haloarchaea in three, geographically distant, Australian saltern crystallizer ponds Extremophiles 2010 14 161 169 10.1007/s00792-009-0295-6 20091074 PMC2832888

[B16] Zafrilla B Martinez-Espinosa RM Alonso MA Bonete MJ Biodiversity of Archaea and floral of two inland saltern ecosystems in the Alto Vinalopó Valley, Spain Saline Syst 2010 6 10 10.1186/1746-1448-6-10 20942947 PMC2984398

[B17] Minegishi H Mizuki T Echigo A Fukushima T Kamekura M Usami R Acidophilic haloarchaeal strains are isolated from various solar salts Saline Syst 2008 4 16 10.1186/1746-1448-4-16 18957135 PMC2583988

[B18] Minegishi H Echigo A Nagaoka S Kamekura M Usami R Halarchaeum acidiphilum gen. nov., sp. nov., a moderately acidophilic haloarchaeon isolated from commercial solar salt Int J Syst Evol Microbiol 2010 60 2513 2516 10.1099/ijs.0.013722-0 19965997

[B19] Roh SW Bae JW Halorubrum cibi sp. nov., an extremely halophilic archaeon from salt-fermented seafood J Microbiol 2009 47 162 166 10.1007/s12275-009-0016-y 19412599

[B20] DasSarma S DasSarma P Halophiles, Encyclopedia of life Sciences 2006 Wiley, London

[B21] DasSarma P Coker JA Huse V DasSarma S Flickinger MC Halophiles, industrial applications Encyclopedia of industrial biotechnology: bioprocess, bioseparation and cell technology 2010 John Wiley & Sons, Inc, Hoboken, NJ

[B22] McGenity TJ Gemmell RT Grant WD Stan-Lotter H Origins of halophilic microorganisms in ancient salt deposits Environ Microbiol 2000 2 243 250 10.1046/j.1462-2920.2000.00105.x 11200425

[B23] Mormile MR Biesen MA Gutierrez MC Ventosa A Pavlovich JB Onstott TC Fredrickson JK Isolation of Halobacterium salinarum retrieved directly from halite brine inclusions Environ Microbiol 2003 5 1094 1102 10.1046/j.1462-2920.2003.00509.x 14641589

[B24] Williams WD Conductivity and salinity of Australian salt lakes Aust J Mar Fresh Res 1986 37 177 182 10.1071/MF9860177

[B25] Braganca JM Furtado I Isolation and characterization of Haloarchaea from low-salinity coastal sediments and waters of Goa Curr Sci 2009 96 1182 1184

[B26] Elevi R Assa P Birbir M Ogan A Oren A Characterization of extremely halophilic Archaea isolated from the Ayvalik Saltern, Turkey World J Microbiol Biotechnol 2004 20 719 725 10.1007/s11274-004-4515-z

[B27] Salgaonkar BB Kabilan M Braganca JM Sensitivity of Haloarchaea to eubacterial pigments produced by Pseudomonas aeruginosa SB1 World J Microbiol Biotechnol 2011 27 799 804 10.1007/s11274-010-0519-z

[B28] Raghavan TM Furtado I Expression of carotenoid pigments of haloarchaeal cultures exposed to aniline Environ Toxicol 2005 20 165 169 10.1002/tox.20091 15793825

[B29] Litchfield CD Irby A Kis-Papo T Oren A Comparisons of the polar lipid profiles of two solar salterns located in Newark, California, U.S.A., and Eilat, Israel Extremophiles 2000 4 259 265 10.1007/s007920070011 11057909

[B30] Oren A Duker S Ritter S The polar lipid composition of Walsby’s square bacterium FEMS Microbiol Lett 1996 138 135 140 10.1111/j.1574-6968.1996.tb08146.x

[B31] Fukushima TT Usami R Kamekura M A traditional Japanese-style salt field is a niche for haloarchaeal strains that can survive in 0.5% salt solution Saline Syst 2007 3 2 10.1186/1746-1448-3-2 17346353 PMC1828056

[B32] Birbir M Calli B Mertoglu B Bardavid RE Oren A Ogmen MN Ogen A Extremely halophilic Archaea from Tuz Lake, Turkey and the adjacent Kaldirim and Kayacik salterns World J Microbiol Biotechnol 2007 23 309 316 10.1007/s11274-006-9223-4

[B33] Tamura K Peterson D Peterson N Stecher G Nei M Kumar S MEGA5: Molecular Evolutionary Genetics Analysis using maximum likelihood, evolutionary distance, and maximum parsimony methods Mol Biol Evol 2011 28 2731 2739 10.1093/molbev/msr121 21546353 PMC3203626

[B34] Wright AG Phylogenetic relationships within the order Halobacteriales inferred from 16S rRNA gene sequences Int J Syst Evol Microbiol 2006 56 1223 1227 10.1099/ijs.0.63776-0 16738095

[B35] Stan-Lotter H Pfaffenhuemer M Legat A Busse HJ Radax C Gruber C Halococcus dombrowskii sp. nov., an archaeal isolate from a Permian alpine salt deposit Int J Syst Evol Microbiol 2002 52 1807 1814 10.1099/ijs.0.02278-0 12361290

[B36] Wang QF Yang WLH Liu YL Cao HH Pfaffenhuemer M Stan-Lotter H Guo GQ Halococcus qingdaonensis sp. nov., a halophilic archaeon isolated from a crude sea-salt sample Int J Syst Evol Microbiol 2007 57 600 604 10.1099/ijs.0.64673-0 17329792 PMC3182530

[B37] Oren A Arahal DR Ventosa A Emended descriptions of genera of the family Halobacteriaceae Int J Syst Evol Microbiol 2009 59 637 642 10.1099/ijs.0.008904-0 19244452

[B38] Elshahed MS Najar FZ Roe BA Oren A Dewers TA Krumholz LR Survey of archaeal diversity reveals an abundance of Halophilic Archaea in a low-salt, sulfide- and sulfur-rich spring Appl Environ Microbiol 2004 70 2230 2239 10.1128/AEM.70.4.2230-2239.2004 15066817 PMC383155

[B39] Pinar G Saiz-Jimenez C Schabereiter-Gunter C Blanco-Valera TM Lubitz W Rolleke S Archaeal communities in two disparate deteriorated ancient wall paintings: detection, identification, and temporal monitoring by denaturing gradient gel electrophoresis FEMS Microbiol Ecol 2001 37 45 54

[B40] Purdy KJ Cresswell-Maynard TD Nedwell DB McGenity TJ Grant WD Timmis KN Embley TM Isolation of haloarchaea that grow at low salinities Environ Microbiol 2004 6 591 595 10.1111/j.1462-2920.2004.00592.x 15142247

[B41] Rölleke S Witte A Wanner G Lubitz W Medieval wall painting– a habitat for archaea: Identification of archaea by denaturing gradient gel electrophoresis (DGGE) of PCR amplified gene fragments coding for 16S rRNA in a medieval wall painting Int Biodeter Biodegr 1998 41 85 92 10.1016/S0964-8305(98)80011-5

[B42] Benlloch S López-López A Casamayor EO Øvreås L Goddard V Daae FL Smerdon G Massana R Joint I Thingstad F Pedrós Alió C Rodríguez-Valera F Prokaryotic genetic diversity throughout the salinity gradient of a coastal solar saltern Environ Microb 2002 4 349 360 10.1046/j.1462-2920.2002.00306.x 12071980

[B43] Jiang H Dong H Yu B Liu X Li Y Ji S Zhang CL Microbial response to salinity change in Lake Chaka, a hypersaline lake on Tibetan plateau Environ Microbiol 2007 9 2603 2621 10.1111/j.1462-2920.2007.01377.x 17803783

[B44] Norton CF Grant WD Survival of halobacteria within fluid inclusions in salt crystals J Gen Microbiol 1988 134 1365 1373

[B45] Özcan B Özcengiz G Colleri A Cokmus C Diversity of halophilic Archaea from six hypersaline environments in Turkey J Microbiol Biotech 2007 17 985 992 18050917

[B46] Pan HL Zhou C Wang HL Xue YF Ma YH Diversity of halophilic archaea in hypersaline lakes of Inner Mongolia, China Acta Microbiol Sin 2006 46 1 6 16579455

[B47] Benlloch S Acinas SG Martínez-Murcia AJ Rodríguez-Valera F Description of prokaryotic biodiversity along the salinity gradient of a multipond solar saltern by direct PCR amplification of 16S rDNA Hydrobiologia 1996 329 19 31 10.1007/BF00034544

[B48] Antón J Roselló-Mora R Rodríguez-Valera F Amann RI Extremely halophilic Bacteria in crystallizer ponds from solar salterns Appl Environ Microbiol 2000 66 3052 3057 10.1128/AEM.66.7.3052-3057.2000 10877805 PMC92110

[B49] Munson MA Nedwell DB Embley TM Phylogenetic diversity of archaea in sediment samples from a coastal salt marsh Appl Envion Microbiol 1997 63 4729 4733 10.1128/aem.63.12.4729-4733.1997 PMC168796 9406392

[B50] Burns DG Camakaris HM Janssen PH Dyall-Smith ML Combined use of cultivation-dependent and cultivation-independent methods indicates that members of most haloarchaeal groups in an Australian Crystallizer pond are cultivable Appl Environ Microbiol 2004 70 5258 5265 10.1128/AEM.70.9.5258-5265.2004 15345408 PMC520848

