# Supplementary material for: Culturable halophilic archaea at the initial and crystallization stages of salt production in a natural solar saltern of Goa, India
Source: Aquat Biosyst. 2012 Jun 29;8:15. doi: 10.1186/2046-9063-8-15 (PMC3444409; doi:10.1186/2046-9063-8-15)
Supplement: Additional file 1 — Figure S1. Solar Salt production at the Ribandar salterns a) bed preparative stage showing series of rectangular beds (January) b) Sluice gate / inlet point for entry of saline water c-d) rectangular beds inundated with saline water (February - March) e) tool used for extracting salt f) crude salt heaped up at the corners of the bandhs g) collection of brine sample h) crude salt collected and piled up on the sides i) collection of sediment sample. Figure S2. Media plates (NTYE and NT) showing diversity of halophilic microorganisms obtained during initial stage and crystallization stages of salt production. Interested bright orange culture obtained on media containing ampicillin. [file 2046-9063-8-15-S1.doc]

**
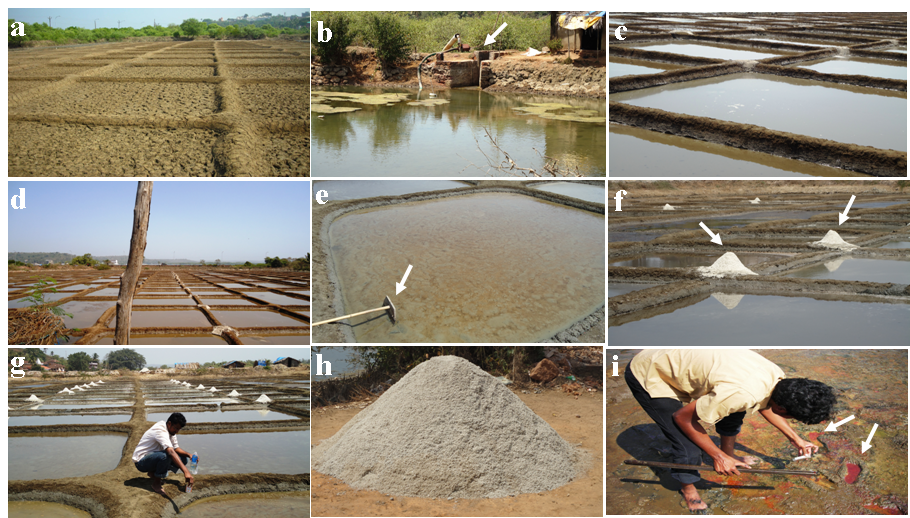
**

**Figure 1 Solar Salt production at the Ribandar salterns a) bed preparative stage showing series of rectangular beds (January) b) Sluice gate / inlet point for entry of saline water c-d) rectangular beds inundated with saline water (Febrruary - March) e) tool used for extracting salt f) crude salt heaped up at the corners of the *bandhs* g) collection of brine sample h) crude salt collected and piled up on the sides i) collection of sediment sample.**


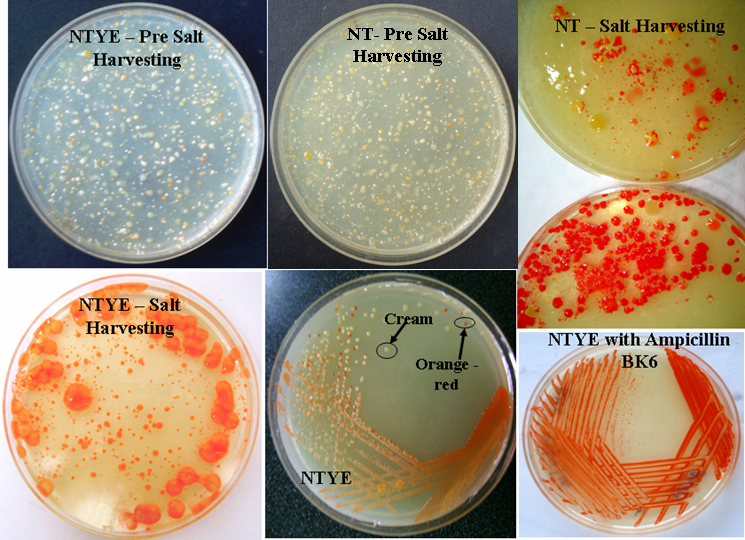


**Figure 2 Media plates (NTYE and NT) showing diversity of halophilic microorganisms obtained during initial stage and crystallization stages of salt production. Interested bright orange culture obtained on media containing ampicillin which got rid of the accompanying white cream culture.**
